# Long term outcomes in survivors of epidemic Influenza A (H7N9) virus infection

**DOI:** 10.1038/s41598-017-17497-6

**Published:** 2017-12-08

**Authors:** Jiajia Chen, Jie Wu, Shaorui Hao, Meifang Yang, Xiaoqing Lu, Xiaoxiao Chen, Lanjuan Li

**Affiliations:** 1 State Key Laboratory for Diagnosis and Treatment of Infectious Diseases, The First Affiliated Hospital of College of Medicine, Zhejiang University, 79 Qingchun Road, Hangzhou Zhejiang, China; 2 Collaborative Innovation Center for Diagnosis and Treatment of Infectious Diseases, Hangzhou, China

## Abstract

Patients who survive influenza A (H7N9) virus infection are at risk of physical and psychological complications of lung injury and multi-organ dysfunction. However, there were no prospectively individualized assessments of physiological, functional and quality-of-life measures after hospital discharge. The current study aims to assess the main determinants of functional disability of these patients during the follow-up. Fifty-six influenza A (H7N9) survivors were investigated during the 2-year after discharge from the hospital. Results show interstitial change and fibrosis on pulmonary imaging remained 6 months after hospital discharge. Both ventilation and diffusion dysfunction improved, but restrictive and obstructive patterns on ventilation function test persisted throughout the follow-up period. For patients with acute respiratory distress syndrome lung functions improved faster during the first six months. Role-physical and Role-emotional domains in the 36-Item Short-Form Health Survey were worse than those of a sex- and age-matched general population group. The quality of life of survivors with ARDS was lower than those with no ARDS. Our findings suggest that pulmonary function and imaging findings improved during the first 6 months especially for those with ARDS, however long-term lung disability and psychological impairment in H7N9 survivors persisted at 2 years after discharge from the hospital.

## Introduction

During the spring of 2013, a novel avian-origin influenza virus emerged. This new virus had a genome similar phylogenetically to that of a chicken A(H7N9) virus isolated from an epidemiologically linked live poultry market^1^ and was thus identified as an avian (H7N9) virus^1–3^. H7N9 viruses can cause severe illnesses in persons with contact to poultry, including pneumonia and acute respiratory distress syndrome (ARDS) with high case fatality rates^2,4^. As of August 31, 2016, a total of 795 laboratory-confirmed cases of human infection with avian influenza A(H7N9) virus had been reported in China^4^. The infections were also detected in the travelers of Canada (two) and Malaysia (one) to China^4^. Although the clinical features of hospitalized patients with H7N9 virus infection are generally similar to those of patients with severe pandemic H1N1^5^ or H5N1 virus infections^6^, the mortality rates of H7N9 and H5N1 have been reported to be 37.1% and 53.2%, respectively^7^, whereas that of H1N1 was <1%^5^.

Patients who survive influenza A (H7N9) virus infection are at risk of physical and psychological complications of lung injury and multi-organ dysfunction. However, previous studies have not included prospectively individualized assessments of physiological, functional and quality-of-life measures after hospital discharge to assess the main determinants of functional disability. Therefore, the goal of this study was to assess the long-term changes in pulmonary function and quality of life among patients recovering from H7N9 infection.

## Results

**Figure 1:**
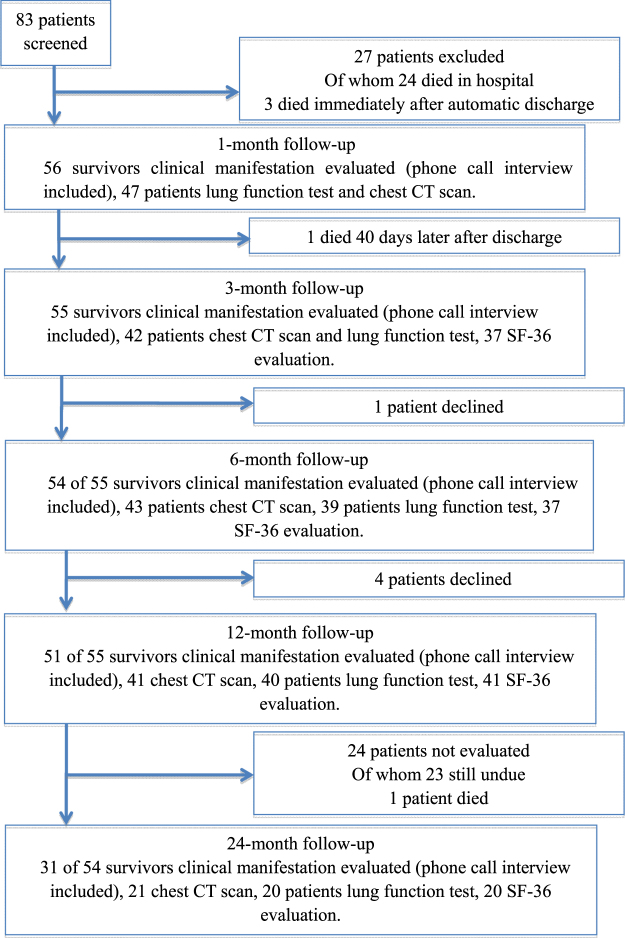
Enrollment of patients with H7N9 infection and follow-up for 2-year after discharge from hospital.

Of the 83 patients with H7N9 infection between March 2013 and May 2014, 27 (33.7%) died in the hospital or immediately after discharge. There were a total of 56 patients were enrolled, and the median follow-up interval was 565 ± 158 days. Sixteen (16/56, 28.6%), 30 (30/55, 54.5%), 44 (44/55, 80.0%) and 48 (48/55, 87.2%) patients returned to work within 1, 3, 6 and 12 months, respectively. Two patients died after discharge and the 2-year mortality rate was 3.6%. One patient died of renal failure and one patient died of pancreatic carcinoma which was diagnosed before H7N9 influenza attack. The enrollment process is outlined in Fig. 1.

### Chest radiography

Chest radiography indicated ground-glass opacities and consolidation at the onset of disease, with the exception of 8.9% (5/56) that showed minor changes. Radiologic changes included linear fibrosis, isolated areas of pleural thickening, and small bullous cysts on CCT at 3 months. At 6 months after discharge from the hospital, all patients showed improvement on CCT; however, no marked change was evident after 6 months. At the 12-month follow-up, 14.6% (6/41) of patients were proximally normal, 41.5% (17/41) had fibrosis and 51.2% (21/41) had parenchymal opacification including ground-glass opacities (GGO) and reticular patterns. Imaging abnormalities including bronchiectasis (n = 10; 24.4%), pneumatocele (n = 4; 9.8%), small bullous cysts (n = 2; 4.9%), nodules (n = 4; 9.8%), and pleural thickening (n = 9; 22.0%) were also identified.

**Figure 2:**
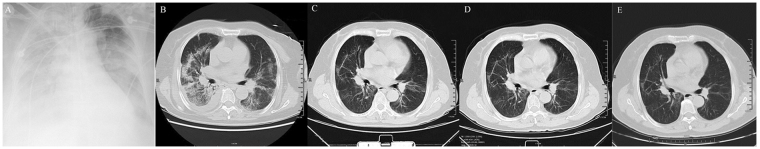
Radiologic findings of 67-year-old female patient with severe avian H7N9 infections between admission and 1-year follow-up. (**A**) Initial bedside chest X-ray image showed white lung on the right side on day 13 after the onset of illness. (**B**) High-resolution CT (HRCT) scan obtained 40 days after disease onset still showed ground-glass opacities (GGOs), multifocal consolidation and pleural effusion. (**C**) At the 3-month visit from discharge, the same scan as B showed GGOs still presented. Consolidation and pleural effusion disappeared. Reticular pattern changes and bronchiectasis were seen. (**D**) and (**E**) At 6-month (**D**) and 12-month (**E**) visit, GGOs and fibrosis can still be seen but much improved on the same scan level as B.

The radiologic findings of a 67-year-old female patient with hypertension were monitored from admission until the 12-month follow-up visit (Fig. 2).

### Lung function

Forty seven of them were included in the analysis of the index of lung function. Of the 47 patients, 20 were diagnosed with ARDS. Their first visit’s clinical and laboratory features were compared and summarized in Table 1. The proportion of female gender was similar between the patients with ARDS and those without ARDS. However, the patients with ARDS were significantly older than those without ARDS. Similarly, patients with ARDS had higher reported acute physiology and chronic health evaluation II scores (APACH II) than the patients without ARDS. ARDS patients tended to stay longer in the hospital than non-ARDS patients. Overall, lung function at the 1-month visit was better in patients without ARDS than in those with ARDS.

**Table 1:** Characteristics of the patients with H7N9 infections in hospital and at 1-month follow-up (n = 47) (m ± SD).

Characteristics	ARDS (n = 20)	Non-ARDS (n = 27)	P value
Age, years	60.8 ± 14.1	50.4 ± 12.6	**0.02006**
Gender, female	9 (45%)	10(37%)	0.7645
APACHE П score	21.0 ± 4.6	12.7 ± 4.6	**p** < **0.01**
Smoking history, yes	4 (20%)	7 (27%)	0.7324
Smoking pack years	1.3 ± 3.6	7.1 ± 14.7	0.092
Morbidity, yes	12 (60%)	11(42%)	0.3726
ECMO, yes	6 (33%)	0	0.05683
Corticosteroid using in hospital, yes	11 (55%)	8 (30%)	0.1347
Type of corticosteroid	Methylprednisolone	Methylprednisolone	—
Dosages of corticosteroid (mg)	459 ± 238	283 ± 82	0.062
Hemopurification, yes	11 (55%)	2 (7%)	**0.01508**
Hospital stay, days	33.1 ± 17.1	13.1 ± 7.7	**p** < **0.01**
Clinical test in hospital			
LDH (IU/L)	545.5 ± 173.1	473.7 ± 331.2	0.3477
Lymphocyte count (×10^9^/L)	0.41 ± 0.17	0.68 ± 0.31	**p** < **0.01**
peak CRP (mg/dL)	159.4 ± 82.4	82.8 ± 75.2	**p** < **0.01**
Lung function at 1-month follow-up			
FEV1(%)	65.3 ± 17.2	82.8 ± 17.6	**p** < **0.01**
FVC(%)	58.2 ± 16.6	81.6 ± 19.5	**p** < **0.01**
DLCO(%)	44.3 ± 17.6	67.7 ± 23.6	**0.01572**
FEV1/FVC(%)	103.1 ± 10.9	117.9 ± 20.9	**0.007651**

ARDS: acute respiratory distress syndrome; APACHE П: acute physiology and chronic health evaluation П; ECMO, extracorporeal membrane oxygenation; LDH: lactate dehydrogenase; L CRP: c-reaction protein; FEV1: forced expiratory volume in one second; FVC: forced vital capacity; FEV1/FVC: the ratio of forced expiratory volume in one second to forced vital capacity; DLCO: carbon monoxide diffusion capacity.

Both ventilation and diffusion dysfunction persisted throughout the follow up. The percentage of ventilation dysfunction in patients decreased from the first visit to the 24-month follow-up visit. The percentages of ventilation dysfunction were 78.7% (37/47), 61.9% (26/42), 61.5% (24/39), 47.5% (19/40) and 55.0% (11/20) at 1,3,6,12 and 24 months, respectively (*p*_*trend* = _0.005). The restrictive ventilation dysfunction ratio decreased from 31.9% (15/47) to 10.0% (2/20) at the 24-month visit (*p* > 0.05). The hybrid ventilation ratio decreased significantly from 29.8% (14/47) to 7.5% (3/20) (*p* < 0.05). The percentage of small airway dysfunction and obstructive patterns tended to increase, albeit not significantly.

Abnormal diffusing capacity of the lungs for carbon monoxide (DLCO) persisted in 92.6% (25/27), 70.5% (12/17), 65.5% (19/29), 78.9% (30/38) and 77.8% (14/18) of the patients by 1, 3, 6, 12 and 24 months, respectively (*p*_*trend* = _0.2894). Most patients had a mild-to-moderate reduction in the severity of DLCO impairment.

### The influence of ARDS on lung function during follow-up

**Figure 3:**
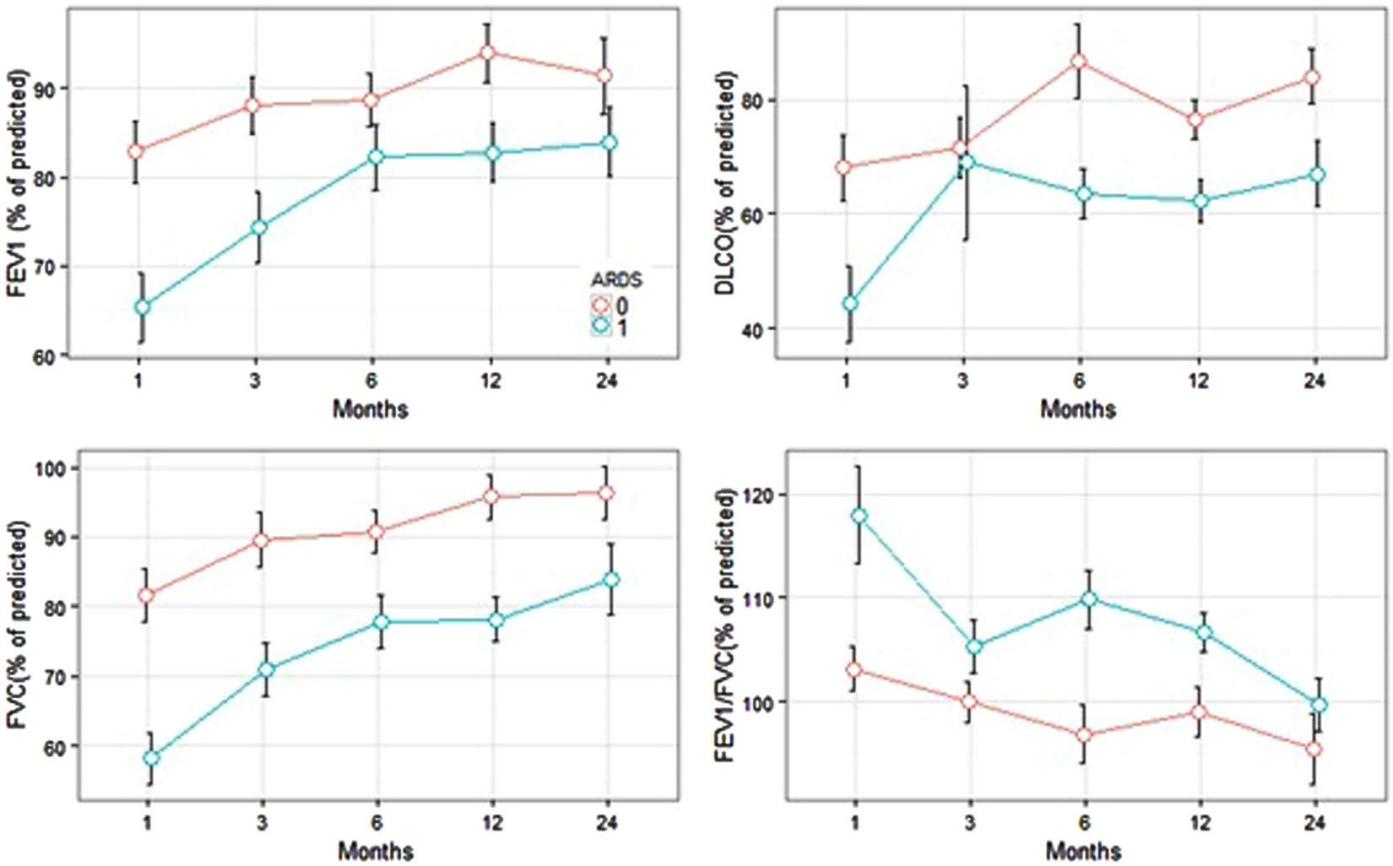
The influence of ARDS on lung function changes of the survivors with H7N9 infections during follow up.

The mean and 95% CI of parameters of lung function over time are plotted in Fig. 3. Estimated longitudinal effects on lung function from the mixed-effects regression models are shown in Table 2. We observed general increases in forced expiratory volume in one second (FEV1), DLCO and forced vital capacity (FVC) for patients regardless of ARDS status. However, patients without ARDS consistently achieved higher FEV1, DLCO and FVC scores over the study period (Fig. 3, Table 2). The ratio of forced expiratory volume in one second to forced vital capacity score (FEV1/FVC) declined over the follow-up period, and was higher in ARDS patients than patients without ARDS (Fig. 3, Table 2). The estimated improvement in FEV1 for ARDS patients was10.54 (*p* = 0.00133), 16.28 (*p* < 0.01), 17.80 (*p < *0.01), and 20.64 (*p* < 0.01) at the 3-, 6-, 12- and 24-month follow-up assessments compared to 1-month follow-up, respectively. For non-ARDS patients, the estimated improvement in FEV1 was smaller (Table 2). The results were similar for other measures, such as FVC, DLCO, and FEV1/FEV.

**Table 2:** Results from mixed-effect regression models of the influence of ARDS on the patients with H7N9 infection.

Parameter	FEV1		FVC		FEV1/FVC		DLCO	
	Est.	p	Est.	p	Est.	p	Est.	p
Main effects								
ARDS		**<**0.01		**<**0.01		**<**0.01		0.0233
Visit		**<**0.01		**<**0.01		0.0501		**<**0.01
Interaction		0.045		0.14		0.1893		0.8336
ARDS*Visit								
Change = 1-month Follow-up								
No ARDS								
3 months	4.2745	0.8828	6.68851	0.3166	−3.5196	0.9643	5.9225	0.9887
6 months	8.6847	0.0759	10.7811	**0.0059**	−5.2527	0.7125	24.2705	**<0.01**
12 months	12.047	**<0.01**	14.4027	**<0.001**	−3.8862	0.9377	14.3968	0.1137
24 months	7.3071	0.5886	12.3483	**0.0218**	−7.1335	0.6221	18.906	0.0667
ARDS								
3 months	10.541	**0.0133**	12.479	**<0.01**	−11.6019	**<0.01**	28.2033	**0.0359**
6 months	16.282	**<0.01**	17.5496	**<0.01**	−7.2176	0.3481	27.0797	**<0.01**
12 months	17.801	**<0.01**	18.5572	**<0.01**	−10.3128	**0.0205**	26.4752	**<0.01**
24 months	20.635	**<0.01**	23.0783	**<0.01**	−16.1209	**<0.01**	29.4434	**0.0134**

ARDS: acute respiratory distress syndrome; FEV1: forced expiratory volume in one second; FVC: forced vital capacity; FEV1/FVC: the ratio of forced expiratory volume in one second to forced vital capacity; DLCO: carbon monoxide diffusion capacity; est: estimated.

### Quality of life

**Figure 4:**
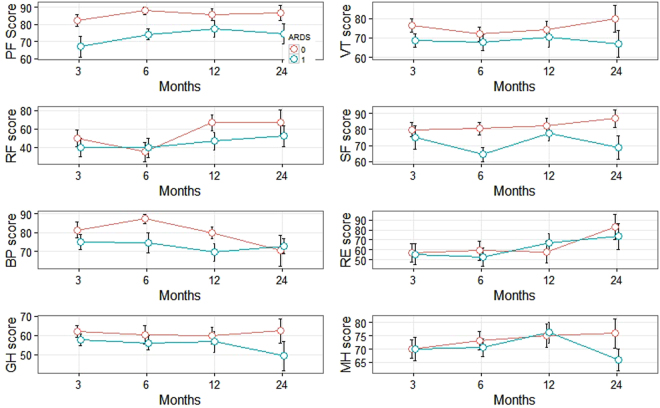
The influence of ARDS on Health-Related Quality of Life over time.

The scores for all domains of the SF-36 did not change significantly from 3 to 24 months after discharge from the hospital. Because the patients were residents in and near Hangzhou, so we chose the SF-36 results of the residents in Hangzhou as the control surveyed by Wang, Li *et al*.^9^. The scores for role-physical (RP) and role-emotional (RE) domains were significantly lower than those of the control population during the first year^9^. RP remained lower than that of the controls, but there was no difference in RE at the 24-month follow-up. Social functioning (SF) and body pain (BP) were both lower than those of the controls; a significant difference was detected in the former at the 6-month follow-up and in the latter at the 12- and 24-month follow-up visits (Table 3). The mean and 95% CI of parameters of quality of life over time are plotted in Fig. 4. Generally, patients with no ARDS reported higher scores on all the domains of quality of life except for RE, which were comparable between patients with ARDS and patients without ARDS across the study period.

**Table 3:** Health-Related Quality of Life among survivors with H7N9 infections during the first 24 months after discharge from the hospital. (M ± SD).

SF-36 Domains	Normal*	3 Months (n = 37)†	6 Months (n = 37)‡	12 Months (n = 41)¶	24 Months (n = 20) §
PF	82.2 ± 19.8	76.0 ± 20.3(0.074)	80.9 ± 13.7(0.581)	81.4 ± 19.0(0.796)	80.0 ± 17.5(0.580)
RP	81.2 ± 33.6	45.9 ± 40.4(0.000)	37.2 ± 44.3(0.000)	55.6 ± 41.8(0.000)	58.8 ± 40.0(0.021)
BP	81.5 ± 20.5	78.8 ± 17.6(0.363)	80.7 ± 19.1(0.805)	74.3 ± 19.3(0.022)	71.8 ± 17.6(0.031)
GH	56.7 ± 20.2	60.3 ± 12.3(0.091)	58.4 ± 18.4(0.589)	58.1 ± 22.0(0.698)	55.3 ± 22.0(0.787)
VT	52.0 ± 20.9	73.2 ± 15.8**(0.000)	69.7 ± 16.8**(0.000)	72.2 ± 20.9**(0.000)	72.8 ± 21.3**(0.001)
SF	83.0 ± 17.8	77.8 ± 23.4(0.188)	72.3 ± 19.4**(0.002)	79.9 ± 20.9 (0.344)	76.8 ± 21.6(0.240)
RE	84.4 ± 32.4	56.5 ± 41.3**(0.000)	55.9 ± 39.3**(0.000)	62.6 ± 46.7**(0.005)	77.8 ± 37.9(0.469)
MH	59.7 ± 22.7	70.1 ± 16.0**(0.000)	71.8 ± 14.7**(0.000)	75.7 ± 19.0**(0.000)	70.4 ± 14.6**(0.006)

PF, physical functioning;SF, social functioning;RP, physical role;RE, emotional role; MH,mental health;BP, body pain; VT, vitality;GH,general health.

*normal valures were calculated in an-age and sex-matched population according to the study of HM Wang, Lu Li and YI Sheng.

P value was listed in the brackets comparing to the normal index espectively.**p < 0.05

‡By 6 months,43 attended the face-to-face interview, and 6 patients refused to do the evaluation.

†By 3 months, 42 patients attended the face-to-face interview, and 37 patients underwent the SF-36 by 3 months, five patients refused to do the evaluation.

¶By 12 months, 41 atternded the face-ro-face interview and do the evaluation.

§By 24 months, 21 patients atternded the face-ro-face interview and one refused to do the evaluation.

## Discussion

Hospitalized patients with H7N9 virus infection usually present with fever and cough, with early sputum production, and the illness progresses rapidly to severe pneumonia, moderate-to-severe ARDS, and shock. The development of refractory hypoxemia is the usual cause of death^2^. However, there are no previous reports on the quality of life of H7N9 patients after hospital discharge. Our study found that more than half of the survivors of H7N9 virus infection had respiratory tract manifestations after discharge from the hospital. Most symptoms improved within 1 month (data not shown). Six months after discharge, more than 80% of patients had returned to work, and the percentage of abnormal DLCO was lowest. Psychological impairment persisted throughout the follow-up period.

All survivors were found to have lung involvement on HRCT images, possibly due to diffuse alveolar damage with proteinaceous exudates, occasional cytomegaly, and intra-alveolar hemorrhage^10^. Imaging showed improvement in inflammation over time, especially during the first 6 months after hospital discharge. However, no further significant changes in interstitial fibrosis or ground-glass opacities were detected at the 12- and 24-month visits. An autopsy study of patients with H7N9 infection suggested that lung histology varied according to the duration of illness. After acute diffuse alveolar damage, post-inflammatory changes such as pulmonary pneumocyte hyperplasia and parenchyma fibroproliferation occurred during the later course of the disease^10^. We speculate that changes during the 6-month convalescence period are irreversible. Absorption occurred slowly and was coincident with clinical symptoms. In survivors of H5N1 virus infection, radiologic abnormalities including ground-glass opacities with a reticular pattern remained evident at the 12-month follow-up visit^10^. Moreover, in a study of the long-term outcomes of pandemic 2009 H1N1-associated severe ARDS, the patients also had abnormal imaging findings, with mildly distorted septal lines, parenchymal bands, pneumatocele and distal bronchiectasis, at 1 year post-ICU discharge^11^. At the 3-month visit, ground-glass opacities were evident in 85.7% of patients^5^. These features are generally similar to those of survivors of H7N9 infection in this study.

Fibrosis (41.5%) and parenchymal pacifications (51.2%), which paralleled lung dysfunction, were common at the 1-year visit. Parenchymal pacifications were more sensitive than CT imaging in the evaluation of fibrotic changes^12^. Pulmonary function has been reported to be near normal, with the exception of decreased diffusion capacity, in H1N1 patients^11^. In our study, approximately half of the survivors had ventilation dysfunction at 24 months. Hybrid patterns and restrictive ventilation dysfunction accounted for most types of dysfunction, which may be caused by muscle weakness and fatigue^13^. 78.9% of patients exhibited decreased DLCO levels at the 1-year follow-up visit, which was higher than reported previously^14–17^. The overall pattern of lung function impairment suggests impairment in the small airways and the alveolar diffusion pathway.

Furthermore, patients with ARDS had larger lung function changes at each follow-up time. The improvement between 1 month and 6 months after discharge was larger than the improvement between 6 months and 24 months, as was previously reported for ARDS^18^. For example, patients with ARDS achieved 16.28 units of improvement in FEV1 within 6 months, but have only 4.35 units of improvement in the next 18 months. A study of the long-term outcomes of survivors with ARDS reported a mild restrictive pattern on lung-function testing, with a mild-to-moderate reduction in carbon monoxide diffusion capacity at 3 months; The median DLCO improved by 9% of the predicted value from 3 to 12 months^13^. In our study, the median DLCO of the patients with ARDS improved by 11.6–18.4% of the predicted value, which is considerably higher than the rates reported previously.

These survivors stayed a long period of time in the hospital or ICU and suffered from lung injury physically. They also suffered from the fear of death. When they went back home, they not only lacked of activities, but also were isolated by their relatives and neighbors because H7N9 attack made people fear of infection and death. Thus survivors have significantly lower HRQoL than that of the general population and are likely to have social functioning and mental health deficits^19^. Similarly, H7N9 survivors experience persistent HRQoL decrements after discharge. Thus, the disease affected HRQoL mainly in the RP, BP, SF, and RE domains compared with normal controls. A meta-analysis showed that recovery in the HRQoL of ARDS survivors occurred during the first 6 months after discharge^20^, but no significant improvement was evident at the 2-year follow-up in our study. These findings suggest that the quality of life of survivors with ARDS was lower than that of those without ARDS. The severity of the diseases may influence the quality of life the patients.

To our knowledge, this is the first prospective study of the physical and psychological health status of patients with influenza A(H7N9) pneumonia during the convalescent period. This study had several limitations. First, most H7N9 infections occurred in China between 2013 and the present. This was a single-center study involving a limited number of patients over a 1-year period in Zhejiang Province, China. Second, follow-up visits were offered to all patients discharged from the hospital, but some refused to attend and some did not complete follow up. The follow-up rates for LFT were 76%, 71%, 75% and 37% and those for HRQoL were 69%, 78%, 75% and 37% at 3, 6, 12 and 24 months, respectively. Although many indices did not change significantly after 1 year, the study population may not be representative of the entire population of H7N9 survivors. Third, this was a prospective study on the impact of H7N9 on the physical and psychological health of survivors. However, no information on the baseline lung function and quality of life of these patients was available. Although some patients may have underlying pulmonary diseases, most of them received the medicine without further examination. So we cannot compare the index of lung function before and post infection of H7N9. In particular, this group of patients had pre-existing conditions, which may also have affected the HRQoL results. Patients who had suffered acute pathologies reported significant decreases in quality of life, whereas other patients with pre-existing conditions reported significant improvements in terms of reduced BP and improved MH, VT and SF scores^21^. In our study, H7N9 survivors had significantly higher VT and MH scores than the population norms. Thus, those scores may have been higher at baseline, i.e., prior to admission. Finally, after discharge from the hospital, there was no significant improvement; however, whether improvements in physical and mental health would have been detected had the follow-up duration been longer is unknown. Thus, further expanded research is needed.

In summary, long-term lung disability and psychological impairment in H7N9 survivors persisted at 2 years after discharge from the hospital. Pulmonary function and imaging findings improved during the first 6 months especially for those with ARDS. Most survivors returned to work, but at the 2-year follow-up, more than half of survivors still had ventilation and blood-gas diffusion dysfunction. The H7N9 survivors had impaired HRQoL scores that were lower than those of a sex- and age-matched control population, and ARDS substantially influenced these scores.

## Methods

### Study design

The Research Ethics Committee of the First Affiliated Hospital, College of Medicine, Zhejiang University approved the design of this study. Our study is an observational monocentric prospective study. All patients with laboratory-confirmed H7N9 infection admitted to the hospital from March 2013 to May 2014 were enrolled. Verbal consent for follow-up was obtained directly from the patient at the time of discharge.

### Follow-up protocol

Patients were evaluated in clinics at 1, 3, 6, 12 and 24 months after their discharge from the hospital. At each visit, computed tomography of the chest (CCT) and lung function tests (LFT) were performed. The 36-Item Short-Form Health Survey (SF-36) (Chinese version) of the Medical Outcome Study^8^ assessing health-related quality of life (HRQoL) was completed. Patients who declined the face-to-face interview were telephoned to obtain survival information.

### Statistical analysis

Patients’ characteristics were summarized with means ± standard deviation (m ± SD) for continuous variables or with frequency and proportion for categorical variables. Baseline differences in ARDS status were assessed using Student’s t tests, Fisher’s exact tests or chi-square test, whenever is applicable. We plotted the means of lung function and quality of life and the corresponding 95% confidence intervals (CIs) over time to graphically examine the changes in outcomes over time. We estimated mixed-effect models to fit lung function with patients’ ARDS status as the main effect, visit (1,3-,6-,12-, or 24-month follow-up), and the ARDS status-by-visit interaction. The models also included a first-order autoregressive covariance structure to account for repeated measures within each patient. We also assessed the estimated difference in the outcome measures at the 3-,6-,12-, or 24-month follow-up visits compared to those at 1-month visit according to ARDS status through model contrast. The estimated change in lung function relative to 1-month visit was assessed. one sample t tests were used to compare SF-36 scores at the 3-,6-,12-, or 24-month follow-up visits with that of the control group.

### Data availability

The datasets generated during and/or analysed during the current study are available from the corresponding author on reasonable request.

### Ethical approval and informed consent

The study design was approved by The Human Ethics Committee of the First Affiliated Hospital, School of Medicine, Zhejiang University. The methods were carried out in accordance with the relevant guidelines and regulations. Informed consent was obtained from each patient included in the study.
